# Review of "Primate Parasite Ecology: The dynamics and study of host-parasite relationships" by Michael A. Huffman and Colin A. Chapman (Eds.)

**DOI:** 10.1186/1756-3305-2-49

**Published:** 2009-10-16

**Authors:** George Poinar

**Affiliations:** 1 Department of Zoology, Oregon State University, Corvallis, OR 97331, USA

## Abstract

Book review of "Primate Parasite Ecology: The dynamics and study of host-parasite relationships" by Michael A. Huffman and Colin A. Chapman (Eds.)

## Book review

No one can doubt that disease played a major role in human history and still continues to impact human health. Also, many human diseases, such as HIV, Ebola and malaria, have been acquired from our close primate relatives. The potential transmission of infectious agents from monkeys and apes to humans is why the study of primate parasites is so significant. The present work, which is divided into several Parts, consists of 25 chapters authored by one or more of 62 contributors.

Part 1 deals with methods used in studying primate parasite interactions. It begins with a chapter on collecting and diagnosing primate parasites. This is followed with a chapter on extracting and identifying minute nematodes, mostly pin worms, recovered from fecal samples. The next chapter discusses the use of molecular methods for comparing populations of stomach worms (*Oesophagostomum*). This is followed by a discussion on the use of endocrinological analyses to interpret social relationships, anthropogenic disturbances and nutrition of primates. Part 1 ends with a chapter on the use of agent-based modeling to investigate the role played by infectious diseases of primates.

Part 2 covers the natural history and host interactions of primate parasites. The first chapter discusses the behavior of gasterointestinal parasites in relation to host finding and parasite migrations. This is followed by a chapter on the evolution of adaptation and species jumping in primate malaria. Since there are over 30 species of primate *Plasmodium *malaria and at least 5 infect humans, this is an important topic. Whether the association of *P. vivax *and *P. malariae *with humans is ancient or recent is addressed and since both are closely related to New World monkey malaria, a strong case can be made for a transfer from monkey to humans. This scenario is strengthened by the discovery of a fossil *Plasmodium *from the Dominican Republic showing that *Plasmodium *malaria was in the New World 20-30 million years ago.

The next chapter discusses disease avoidance in gorillas and chimpanzees in relation to social organization. The amount of *Ficus*-fruit consumption by the apes is correlated with Ebola outbreaks since the bat reservoir hosts contaminate figs while feeding. This is followed by a review of primate-parasitic zoonoses and anthropozoonoses, including protozoa, nematodes, bot flies (myiasis), fleas, ticks, leeches and trematodes. Included is American trypanosomiasis or Chagas disease, caused by *Trypanosoma cruzi*. Reservoir hosts include rodents, but also probably bats since a member of the latter group carried a *Trypanosoma *very similar to *T. cruzi *20-30 million years ago in Hispaniola. Curiously, the authors omitted reports of simian infections by *Leishmania*, an important human disease in both the New and Old Worlds.

The following chapter discusses the use of primate sucking lice (*Pediculus, Pthrius *and *Pedicinus*) as markers of primate evolutionary history. Pinworms, *Pneumocystis *fungae, *Plasmodium *malaria and simian foamy virus are other agents that could be used as markers for non-human primate evolution.

The following chapter discusses cryptic species (reproductively isolated but morphological indistinguishable) of lice on primates. The human body louse and head louse are considered cryptic species and various methods by which they can be distinguished, including molecular, are discussed. The next chapter discusses the prevalence of *Clostridium perfringens *in the intestine of non-human primates. This bacterium is ubiquitous and a normal component in the guts of many vertebrates. Different rates of infestation occur between wild and captive monkeys and apes. The next chapter discusses the use of molecular sequence analyses to determine differences in numbers of *Clostridium*, *Lactobacillus *and *Bifidobacterium *in wild and captive chimpanzees.

A chapter on gasterointestinal parasites from fecal samples of bonbos (*Pan paniscus*) in Congo's Lomako Forest reports protozoan trophozoites, trematodes and nematodes. This is followed by a chapter on habitat disturbance and seasonal fluctuations of lemur parasites (Sifakas) in Madagascar. Tapeworm and nematode populations were higher in Sifakas (*Propithecus edwardsi*) in disturbed rather than in undisturbed forests. The following chapter questions if climatic differences might influence self-medicative behavior (the folding and swallowing of whole rough, bristly leaves) in gorillas. Apparently this behavior stimulates peristalsis that serves to cleanse the gut of helminths.

The following chapter deals with possible dangers when humans are exposed to simians with novel retroviruses. Apparently a much broader range of simian retroviruses than realized are capable of causing primary zoonotic infections in humans and the authors conclude that transmission is probably occurring continuously. This is followed by a chapter on gasterointestinal parasites infecting *Alouatta *howler monkeys. Whether these infections are natural or were acquired from humans or domestic animals is discussed. The following chapter deals with a possible correlation between social dominance and the distribution of five nematodes in a subspecies of the Japanese macaque, *Macaca fuscata yakai*. The observed aggregated distribution pattern was considered to result from the highly social nature of the monkeys.

Possible differences in the parasite load in wild-feeding and crop raiding baboons were investigated in the next chapter. Significant differences were found regarding some of the parasites. Whether parasites can be a selective force on primate group size is discussed in the following chapter. This hypothesis was tested with red colobus monkeys (*Procolobus rufomitratus*) in Uganda. Results showed no difference in numbers of protozoa and nematodes between large and small monkey troops. The next chapter discussed whether the diet of mountain gorillas in Uganda influenced their nematode and tapeworm parasites. No correlation between parasite load and protein intake was noted, but ingestion of tannin decreased pinworm (*Probstmayria *sp.) infections in females and juveniles. This Part ends with a discussion on host-parasite dynamics and future areas of study in primate-parasite ecology.

This book contains a wealth of information and provides a solid base for future studies of primate-parasite interactions. A higher classification of the primates, down to the species level with those members discussed in the book, would have been a helpful addition.

## Competing interests

The author declares that he has no competing interests.

